# Regulation of neuronal circHomer1 biogenesis by PKA/CREB/ERK-mediated pathways and effects of glutamate and dopamine receptor blockade

**DOI:** 10.21203/rs.3.rs-3547375/v1

**Published:** 2024-01-12

**Authors:** Nikolaos Mellios, Grigorios Papageorgiou, Victor Gorgievski, Gabriella Maxson, Megan Hernandez, Madison Otero, Michael Varangis, Michela Dell’Orco, Nora Perrone-Bizzozero, Eleni Tzavara

**Affiliations:** University of New Mexico HSC; University of New Mexico; University of New Mexico; University of New Mexico; University of New Mexico; University of New Mexico; University of New Mexico; University of New Mexico; University of New Mexico

## Abstract

There are currently only very few efficacious drug treatments for SCZ and BD, none of which can significantly ameliorate cognitive symptoms. Thus, further research is needed in elucidating molecular pathways linked to cognitive function and antipsychotic treatment. Circular RNAs (circRNAs) are stable brain-enriched non-coding RNAs, derived from the covalent back-splicing of precursor mRNA molecules. *CircHomer1* is a neuronal-enriched, activity-dependent circRNA, derived from the precursor of the long *HOMER1B* mRNA isoform, which is significantly downregulated in the prefrontal cortex of subjects with psychosis and is able to regulate cognitive function. Even though its relevance to psychiatric disorders and its role in brain function and synaptic plasticity have been well established, little is known about the molecular mechanisms that underlie *circHomer1* biogenesis in response to neuronal activity and psychiatric drug treatment. Here we suggest that the RNA-binding protein (RBP) FUS positively regulates neuronal *circHomer1* expression. Furthermore, we show that the MEK/ERK and PKA/CREB pathways positively regulate neuronal *circHomer1* expression, as well as promote the transcription of *Fus* and *Eif4a3*, another RBP previously shown to activate *circHomer1* biogenesis. We then demonstrate via both *in vitro* and *in vivo* studies that NMDA and mGluR5 receptors are upstream modulators of *circHomer1* expression. Lastly, we report that *in vivo* D2R antagonism increases *circHomer1* expression, whereas 5HT2AR blockade reduces *circHomer1* levels in multiple brain regions. Taken together, this study allows us to gain novel insights into the molecular circuits that underlie the biogenesis of a psychiatric disease-associated circRNA.

## INTRODUCTION

Non-coding RNAs (ncRNAs) are a diverse group of regulatory RNA molecules that do not encode for proteins^1^. They are involved in a wide range of biological processes, including regulation of gene expression, DNA repair, and the control of cell growth and development, and have been implicated in brain development, function, and psychiatric disease^2–5^. Circular RNAs (circRNAs) are a subclass of ncRNAs that are characterized by a covalently closed loop structure, as opposed to the linear structure of most other RNA molecules^6–8^. They are derived from exons, introns, or intergenic regions, and display robust stability due to their resistance to endonuclease degradation^9–11^. The biogenesis of circRNAs is facilitated by either inverted complementary sequences, in long flanking introns, or by the aid of trans-acting RNA binding proteins (RBPs). CircRNAs are known to play a role in regulating gene expression and have been implicated in a variety of biological processes including cell differentiation, development, and disease. It has been proposed that circRNAs can be preferentially derived from genes that code for synaptic proteins^11–14^. Thus, circRNAs might serve as critical regulators of brain development and function. Recent studies suggest a role of circRNAs in psychiatric disorders. We and others have shown an altered profile of expression of circRNAs in post-mortem brains of SCZ and BP subjects, namely in the cortex a brain region implicated in the pathophysiology of these disorders ^15–17^. Interestingly, among those circRNAs, is *circHomer1*, which regulates the expression of numerous mRNA transcripts from genes involved in synaptic plasticity and psychiatric disease, including *Homer1* itself.

The *Homer1* gene encodes a protein that is a member of the Homer family of scaffold proteins. These proteins play a role in the organization and function of the postsynaptic density (PSD)^18–20^. Homer1 specifically is known to play a role in the clustering and stabilization of ionotropic receptors, such as NMDA receptors at the PSD, as well as in the regulation of receptor trafficking and synaptic plasticity^21–24^. Studies have shown that Homer1 is highly expressed in the brain, particularly in regions involved in cognitive function, such as the frontal cortex. Evidence for the role of *HOMER1* in psychiatric disorders stems from animal studies since *Homer1* KO mice have altered extracellular glutamate levels and display SCZ-like behaviors, while ectopically restoring Homer1 expression can reverse some of these effects^25,26^. Moreover, there is evidence that antipsychotics tightly regulate post-synaptic density (PSD) proteins, including multiple Homer1 isoforms^27–30^. Also, *HOMER1* polymorphisms have been associated with psychiatric disease symptomatology, and recently single nucleotide polymorphisms (SNPs) within the *HOMER1* gene have been included in the list of genome-wide significant genetic loci for both SCZ and BD^31,32^. Furthermore, enough data suggest that an imbalance of the levels of the different *HOMER1* isoforms could have a significant impact on the pathophysiology of psychiatric disorders, such as SCZ and BD^33^.

*CircHomer1* is a brain-enriched circRNA that is derived from exons 2–5 of the precursor of the long *HOMER1B* mRNA isoform (*pre-HOMER1B*)^34^. *CircHomer1* is abundantly expressed in both mouse and human brain, and it is enriched within neurons^34^. Although *circHomer1* is comprised of 4 exons of the linear *pre-HOMER1B* mRNA, it is not protein coding, since it lacks the sequences needed for translation^34^. Previous publications from our group have shown that the levels of *circHomer1* were significantly downregulated in postmortem brain samples of BD and SCZ subjects, in the orbitofrontal cortex (OFC) and dorsolateral prefrontal cortex (DLPFC) respectively. Similarly, a reduced expression of *circHomer1* levels was detected when using iPSC-derived neuronal cultures from SCZ and BD patients^34,35^.

The orbitofrontal cortex (OFC) is a brain region involved in a wide range of cognitive and emotional functions, including decision-making, impulse control, and social behavior, which is also associated with SCZ and BD. Studies in schizophrenia have found that the OFC is structurally and functionally altered in individuals with the disorder^36–38^. Research has also suggested that the OFC may play a key role in the development and maintenance of both the positive symptoms of schizophrenia and disease-related cognitive deficits. Along this line, we showed that in animal models, *circHomer1* knockdown within the OFC disrupted the expression of synaptic plasticity related genes. We provided evidence of an antagonistic interaction between *circHomer1* and *Homer1b* mRNA in the OFC and their differential effects on OFC-mediated cognitive flexibility^34,35^. We also demonstrated that *circHomer1* knockdown within the OFC can affect neuronal firing in vivo and influence OFC-mediated function with regards to reversal learning. Overall, the above findings suggest *circHomer1* as novel target in activity-dependent synaptic plasticity pathways, in brain health and disease. However, no work had been conducted on the mechanisms that underlie the biogenesis of *circHomer1*, and its regulation by cell signaling cascades, neurotransmitters, and drugs relevant to psychiatric disorders.

Here we provide strong evidence that CREB, known to promote gene expression in a neuronal activity-dependent pattern^39^, is a major positive regulator of *circHomer1* biogenesis in neurons, affecting the transcription of *Eif4a3* and *Fus*, genes coding RBPs that promote *circHomer1* biogenesis^35,40^. We show that upstream of CREB, the ERK/MAPK and PKA pathways, which are crucial regulators of synaptic plasticity, are involved in the control of *Eif4a3* and *Fus* expression and subsequently *circHomer1* biogenesis. In addition, we document that *circHomer1* is positively regulated by key-neurotransmitter systems (glutamate, dopamine, serotonin, implicated in SCZ and in antipsychotic drug action) both in vitro and in the brain. We show that NDMAR inactivation can reduce *circHomer1* biogenesis while activation of mGluR5 receptors elevates *circHomer1* levels, especially in the OFC. Finally, we show that serotoninergic 5HT2A or dopaminergic D2 receptor antagonists differentially modulate *circHomer1* expression. Interestingly, olanzapine, a second-generation atypical antipsychotic, with D2 and HT2A antagonist profile regulates *circHomer1* in the OFC but not in subcortical areas.

## MATERIALS AND METHODS

### RNA extraction and mRNA/circRNA quantification.

RNA was isolated using the miRNeasy RNA isolation kit (Qiagen, Hilden, Germany) following the manufacturer’s supplied protocol. RNA quality as well as concentration of isolated total RNA was assayed through Nanodrop 2000 spectrophotometer and Qubit 3 (Thermo Fisher Scientific, Waltham, Massachusetts), with all samples passing the quality control measurements (A260/230 and A260/280). Reverse transcription of total RNA (100–500 nanograms depending on the Nanodrop concentration values) was carried out using the SuperScript IV First-Strand Synthesis System (Thermo Fisher Scientific) with oligo-dT for linear mRNAs and random hexamers for circRNA detection. Quantitative RT-PCR was done using either PowerUp SYBR Green Master Mix (Thermo Fisher Scientific) along with custom designed, validated, and sequence-verified circRNA and mRNA primers or TaqMan Gene Expression Assays (Thermo Fisher Scientific) for mRNA detection. All circRNA qRT-PCR products were run on an agarose gel and sequence validated. At the end of each qPCR amplifications plots and melt curves (ΔRn vs cycle per well) were automatically calculated by Quant Studio 7 Flex. 18S *rRNA* was used as a normalizer for mRNA and circRNA expression levels. For mRNA qRT-PCR quantification the following formula was used: Relative value = ÂCt^*18S rRNA*^/ ÂCt^mRNA^, where A = 10^ (− 1/primer slope). For circRNA qRT-PCR quantification the following formula was used: Relative value = ÂCt^*18S rRNA*^ / ÂCt^circRNA^, where A = 10^ (− 1/primer slope). Details for the primers used (name, species, assay ID, sequence are found in Supplementary Table S1).

### RNA Immunoprecipitation (RIP).

Frontal cortex tissues from adult Wild Type (WT) mice were collected in polysome lysis buffer (50mM Tris-HCl pH 7.4; 150mM NaCl; 0.5% NP-40; 2mM MgCl2; 1mM EDTA, 1mM EGTA, 10mM NaF, 1mM Na3Vo4; 1mM DTT) supplemented with 200 U/mL RNAseOut^®^ Ribonuclease Inhibitor (Invitrogen) and EDTA-free protease inhibitor cocktail (Roche, USA) and disaggregated with a Dounce homogenizer. 40uL Dynabeads (Protein G) (Invitrogen) were aliquoted into microfuge tubes and washed 3 times with 20 μL of PBS 1X at RT. 4 μg of the primary antibodies or IgG controls (listed below) were added in a total volume of 200 μL of 1X PBS. Beads were rotated over-night at 4°C. After washing the beads 3 times NT-2 buffer (50mM Tris-HCl, pH 7.4, 150mM NaCl, 0.05% NP-40, 1mM MgCl2) lysates were added to each tube with Ab-bound beads and rotated at 4°C for 4 hours. Beads were washed with 5 min rotation at 4°C, 6 times with NT-2 buffer supplemented with RNAseOut (100U/mL) and resuspended in 150uL Proteinase K buffer (NT-2 supplemented with 1mg/mL Proteinase K and 1% SDS). Beads were incubated at 55°C, 30 min (vortex occasionally every 5 min); Trizol was added directly to the tubes after Proteinase K treatment for RNA extraction. Aliquots for both RNA and proteins were taken before (input) and after the IP. Primary antibodies: antibody rabbit polyclonal anti-eIF4A3 (A302–981A; Bethyl Laboratories), antibody rabbit polyclonal anti-FUS (NB100–565) Novus Biologicals and anti-HuD (E1, sc-28299; Santa Cruz Biotechnology, Inc.) IgG controls: normal rabbit IgG (sc-2027; Santa Cruz Biotechnology, Inc.); Purified Mouse IgG2a, κ Isotype Ctrl Antibody (#401501; BioLegend). Total RNA was extracted with Trizol^®^ (Invitrogen) according to the manufacturer’s recommendations. RNA quality and quantity were determined using the Qubit (Invitrogen) spectrophotometer. WB analysis was performed by SDS–polyacrylamide gel electrophoresis (PAGE). Extracted proteins were boiled in Laemmli sample buffer (0.6 g/100 mL Tris, 2 g/100 mL SDS, 10% glycerol, 1% β-mercaptoethanol, pH 6.8) for 10 min, separated on 10% SDS-PAGE gel and transferred to a PVDF membrane (Bio-Rad) using a liquid transfer apparatus (Bio-Rad). The membranes were treated with a blocking solution containing 5% non-fat dry milk in TBS-T buffer (10 mM Tris-HCl, 100 mM NaCl, 0.1% Tween, pH 7.5) for 1h and incubated overnight with the primary antibodies. Immunoreactivity was detected using the donkey anti-rabbit (GE Healthcare USA, dilution 1:10,000) or anti-mouse (GE Healthcare USA, dilution 1:10,000) secondary peroxidase-conjugated antibodies. The immunoreactive bands were then visualized using the Western Lightning Plus-ECL (PerkinElmer, Inc).

### SH-SY5y Human neuroblastoma – FUS and GW182 shRNA mediated KDs.

SH-SY5Y epithelial human neuroblastoma cell line was purchased from ATCC CRL-2266^™^. SY-5 can differentiate under certain circumstances. To that end, cells were fed for 5 days using Neurobasal Plus, 1XB27 Plus, 5% Pen/Strep ~ ThermoFisher Scientific. The neuronal-like morphology was observed under the microscope and by measuring the levels of beta-3 tubulin, a neuronal marker. After differentiation, SY-5 cells were plated in a 24 well plate at passage #7 at a concentration of 100,000 cells per well. 48 hours later they were transfected with the shRNA clone for the gene of interest (*FUS and GW182*) and a non-target shRNA clone. Transfections were performed using Lipofectamine^™^ 3000 / P3000 reagent (500ng DNA, 1μl Lipofectamine and 1μl P3000 reagent per well). 48hrs following the transfection, the differentiated SH-SY5Y cells were subjected to RNA extraction and then qRT-PCR to assay overall changes in *circHomer1* expression. For the KD of *FUS* and *GW182*, all shRNAs clones were already predesigned, inserted in a lentiviral shRNA vector (pLKO.1) and purchased from (Millipore Sigma-Aldrich – MISSION^®^ shRNA Products library). Multiple shRNA clones were purchased targeting the CSD region of each gene and after rounds of experiment testing their efficiency, the clone that led to the highest % of KD of *FUS* and *GW182* respectively, was further used to determine changes in *circHomer1* expression levels.

### Primary mouse cortical neurons and the subsequent experiments.

Mouse cortical neuronal cultures were purchased from ThermoFisher Scientific – Catalog number: A15585 (C57BL/6 mice). Neurons were plated at a density of 4–5×10^4 cells/12-mm coverslip coated with poly-Ornithine on a 24-well plate. Primary cortical neuronal cells were allowed to adhere in 20 min before addition of 500ul plating neuronal media. Neurons were fed by replacing half the volume with fresh media every third day (Neurobasal Plus, 1XB27 Plus, 2mM Glutamax, 5% Pen/Strep ~ ThermoFisher Scientific). At DIV13 when neurons were mature enough to form numerous synapses, pharmacological treatments were conducted as mentioned below. Of note, all the agents were dissolved in DMSO and to compensate the effects of DMSO in cells viability, a negative control / vehicle was a treatment group of culture medium along with DMSO at the same concentration ~ 0.1%. 24 hours later following treatment, neurons were subjected to RNA extraction and then qRT-PCR to assay overall changes in *circHomer1* expression.

### The following experiments were performed in primary mouse cortical neurons at DIV13-mature:

#### CREB and CREB-CBP pharmacological inhibition

For CREB inhibition, 666 – 15 (Cat. No. 5661- Tocris Bio-Techne corporation) was added in a dose 50uM < IC50 and follow-up experiment at a dose of 81uM IC50. For inhibiting the interaction of CREB with its co-transcriptional activator CBP, the KIX-KID interaction inhibitor, CBP-CREB Interaction Inhibitor - CAS 92–78–4 – Calbiochem (Millipore Sigma-Aldrich) was added at a concertation of 2.5uM

#### Small molecule kinase inhibitors

To examine which kinase can modulate *circHomer1* levels, various candidates were tested in primary cortical neurons (see protocol above). All agents were added at DIV13 and were purchased from Tocris Bio-Techne corporation. FR 180204 (Cat. No. 3706) was used for ERK inhibition at a dose of 0.3 uM. U0126 (Cat. No. 1144) that inhibits MEK1,2 kinase activity was added at a concentration of 0.06 uM. H 89 dihydrochloride (Cat. No. 2910) that is a selective PKA inhibitor was added at a dose equal to 120nM, while GF 109203X (Cat. No. 0741) which inhibits PKC activity added at a dose equal to 0.2uM.

#### Induction of neuronal activity

To examine the hypothesis that *circHomer1* is activity dependent circRNA, neuronal activity was induced by using a combination of Bicuculine and 4-Aminopyridine. (+)-Bicuculline (Cat. No. 0130 – Tocris Bio-Techne Corporation), that is a potent GABAa antagonist, was added at a dose of 35uM while 4-Aminopyridine (Cat. No. 0940 – Tocris Bio-Techne Corporation), that is a Kv channel blocker, was added at a concentration of 100uM.

#### Glutamatergic receptor modulation

For activating mGluR5, CHPG (Cat. No. 1049 – Tocris Bio-Techne Corporation) a mGluR5 selective agonist was used at a dose of 1mM, whereas for inhibiting NMDAR function, (+)-MK 801 maleate (Cat. No. 0924 Tocris Bio-Techne Corporation) a selective non-competitive antagonist was added at a final concentration of 3uM. Lastly, ANA-12 (Cat. No. 4781 Tocris – BioTechne) that is a TrkB receptor antagonist was added at 1uM.

Pharmacological and psychotropic treatment experiments.

### Animals:

All mice used were C57BL/6J 5-week-old males purchased from Charles River (France). Mice were kept under standard conditions: housed in cage of 5, at 22 ± 1°C, and a 12-h light-dark cycle with food and water available *ad libitum*. Humidity levels were between 45 and 55%. Chronic treatment and behavioral assessments were performed during the second half of the light phase.

All animal protocols and welfare complied with French and European Ethical regulations. The experimental protocols were approved by the local Ethical Committee (Comite d’ ethique en experimentation animale Charles Darwin N°5)

### Drugs and treatments:

Drugs were purchased from Sigma-Aldrich (MDL 100907 and MK801) and Tocris (CDPPB, Olanzapine, Sulpiride).

All agents were dissolved in a vehicle solution (80% saline, 10%DMSO, 10% Cremophor) and were administered i.p. on a daily basis at a volume of 10ml/kg.

At the age of 6-weeks-old, mice were randomly assigned to the respective treatment groups. For each treatment group, 12 mice were used to achieve a statistically significant power.

#### – 1st experiment:

Mice were treated for 14 days with either, a vehicle solution (control group), a selective D2-R antagonist (Sulpiride, 25mg/kg), a selective 5HT-2AR antagonist (MDL100907, 2mg/kg) or an atypical antipsychotic of the 2nd generation (Olanzapine, 20mg/kg).

#### – 2nd experiment:

Mice were treated for 7 days with either, a vehicle solution, a NMDAR antagonist (MK801, 0.3mg/kg), a mGluR5 potentiator (CDPPB, 10mg/kg) or, a combination of both MK801 and CDPPB (0.3mg/kg and 10mg/kg respectively).

### Tissue collection:

#### Mice were terminally anesthetized with ip injection of pentobarbital (200mg/kg in 5% glucose).

Brain was collected and was flash frozen with dry ice. To study gene expression levels after a sustained administration of pharmacological agents, RNA was isolated using the miRNeasy RNA isolation kit (Qiagen, Hilden, Germany). RNA quality and concentration was assayed through Nanodrop 2000 spectrophotometer (ThermoFisher Scientific). Reverse transcription was performed using the SuperScript IV First-Strand Synthesis System (ThermoFisher Scientific) with random hexamers for circRNA and oligo-dT primers for linear mRNA RNA detection. cDNA was then be used together with custom made, validated, and sequence- verified circRNA and mRNA primers or TaqMan mRNA primers (ThermoFisher Scientific) for mRNA qRT-PCR. 18S rRNA was used as a normalizer for mRNA detection, whereas circTulp4 or 18S rRNA was used for circRNA normalization. For qRT-PCR quantification the following formula was used: Relative value = ÂCt^normalizer 18s rRNA^ / ÂCt^mRNA or circRNA^, where A = 10^(−1/primer slope)^.

#### Behavioral experiment: Locomotion.

##### – 1st experiment:

Mice were injected with either, a vehicle solution (control group), a selective D2-R antagonist (Sulpiride, 25mg/kg), a selective 5HT-2AR antagonist (MDL100907, 2mg/kg) or an atypical antipsychotic of the 2nd generation (Olanzapine, 20mg/kg).

Horizontal activity (ambulations) was assessed in transparent activity cages (20 × 15 × 25 cm), with automatic monitoring of photocell beam breaks (actimeter - Imetronic, France). Locomotor activity was recorded, and we conducted analyses between groups, both for 20 min (20 min after the injection) and the total duration of the test.

##### – 2nd experiment:

Mice were pretreated with either a vehicle solution or CDPPB (for the MK801 + CDPPB group), 15 min prior to treatment with either vehicle, MK-801, CDPPB.

Horizontal activity (total distance) was recorded using video tracking for 15min. For video tracking, a CCTV camera (The Imagine Source, Model DMK 22AUC03 with a vari focal 1:1.3/2.8–12mm lens) was placed 80cm above the openfield connected to a computer running ANY-maze software (Version 6.3; Stoelting Co).

### Statistical Analysis.

Normalized values were divided to the mean of each Control group and the relative to control ratios were plotted as means ± S.E.M. using GraphPad Prism and after removing up to 2 outliers using Roots test (Graphpad Software, La Jolla, CA). For the normality and lognormality of the data sets, the following tests were conducted: Anderson-Darling, D’Agostino & Pearson and Shapiro-Wilk test. Due to the fact that the vast majority of datasets passed the tests for normal Gaussian distribution, one sample t test was conducted for comparing 2 groups, while in the few cases where data showed a non-parametric distribution the Wilcoxon Signed Rank test was used instead. For comparisons involving more than 2 groups, a one-way Analysis of Variance (ANOVA) with Tukey’s, Dunnett’s, Bonferroni, or Dunn’s post-hoc correction for multiple comparisons for samples with normal distribution of data and Kruskal-Wallis test with Dunnett’s or Dunn’s post-hoc correction for multiple comparisons was used for samples that did not display normal distribution. Spearman correlation coefficients and two-tailed p-values were calculated. Even though datasets were sampled from a Gaussian distribution, Spearman correlation was used due to the monotonic relationship of the variables and due to the more robust nature of this test for any outliers of the data set.

## RESULTS

### FUS and EIF4A3 regulate circHomer1 biogenesis.

**Figure 1:**
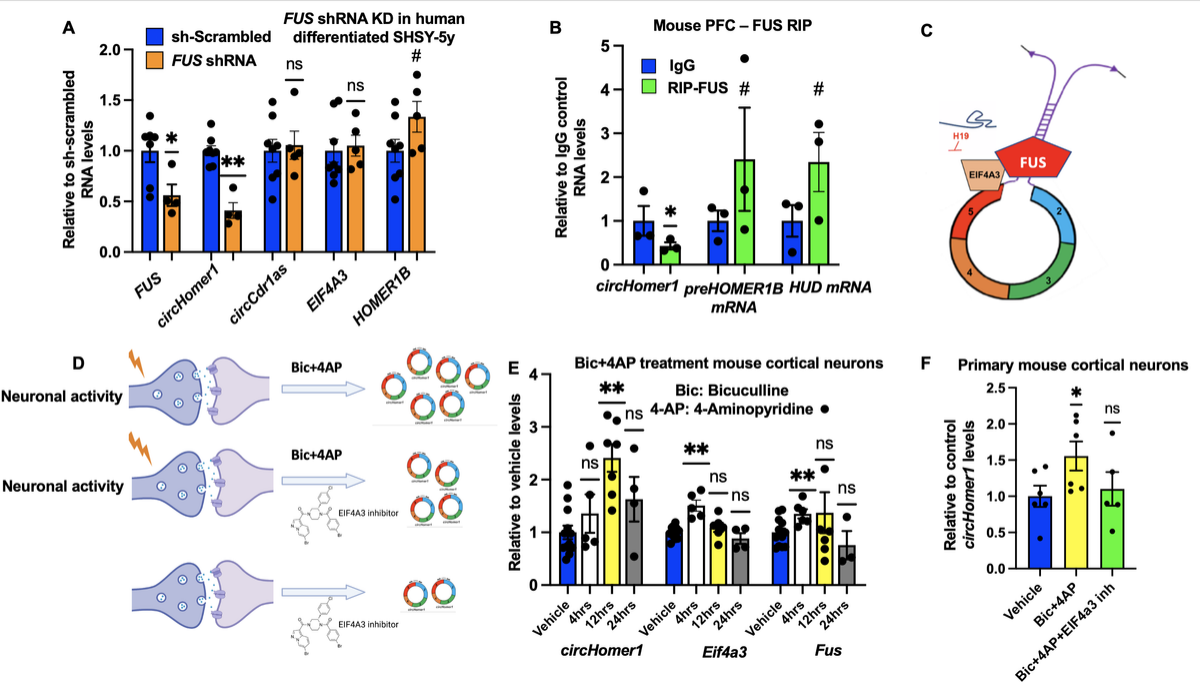
FUS can upregulate *circHomer1* levels while neuronal activity induces *circHomer1*levels. (A) Relative to vehicle *FUS* mRNA, *circHomer1, circCDR1as, EIF4A3*mRNA and *HOMER1B* mRNA levels (based on qRT-PCR and normalized to *18S rRNA*) 48hours after shRNA-mediated knockdown of *FUS* in human differentiated SH-SY5Y cells using the shRNA clone that achieved optimal KD %. Mean ± SEM. *p < 0.05, **p < 0.01, ****p < 0.0001, two tailed one sample t-test relative to shRNA scrambled. For *Fus* in (A): p = 0.0013, t = 4.601, df = 9. For *circHomer1*: p = 0.0047, t = 7.590, df = 7. For *Homer1b*: p = 0.0783, t = 2.353, df = 4. (B) RIP using an antibody against FUS protein. Relative to IgG control, *circHomer1*, *pre-Homer1b mRNA* and *HuD* mRNA levels (based on qRT-PCR and normalized to 18S rRNA) after FUS RIP. Mean ± SEM. #0.10 < p < 0.05, *p < 0.05, one-tailed one sample t-test relative to IgG control levels. For *circHomer1*: p = 0.00945, t=7.167, df=2. For *pre-Homer1b*: p = 0.06095, t = 2.595, df = 2. For *HuD*: p = 0.05685, t = 2.707, df =2. (C) Proposed schematic of *circHomer1* biogenesis: EIF4A3 and FUS can act as upstream regulators promoting *circHomer1* biogenesis, while the long non-coding RNA *H19* can bind to EIF4A3 inhibiting its activity and negatively regulate *circHomer1*. (D) Schematic connecting neuronal activity and EIF4A3 promoted *circHomer1* biogenesis in both basal and neuronal activity conditions. (E) Relative to vehicle *circHomer1, Eif4a3* and *Fus* mRNA levels (quantifications were based on qRT-PCR and normalized to 18S rRNA) after treatment with Bicuculline and 4-Aminopyridine (treatment at different time points 4–12-24 hours) Mean ± SEM. *p < 0.05, **p < 0.01, ****p < 0.0001, two tailed one sample t-test relative to vehicle. In all bar graphs the individual replicates are shown within the graph. For *circHomer1* after 12 hours of treatment: t = 5.369, df = 6, and p = 0.0017. For *Eif4a3* mRNA after 4 hours of treatment: t = 4.801, df = 4, and p = 0.0086. For *Fus* mRNA after 4 hours of treatment: t = 4.079, df = 5, and p = 0.0096. (F) Relative to vehicle, *circHomer1* (quantifications were based on qRT-PCR and normalized to 18S rRNA) after treatment with Bicuculline and 4-Aminopyridine and co-treatment with EIF4A3 inhibitor (treatment was done for 24hours) Mean ± SEM. *p < 0.05, two tailed one sample t-test relative to vehicle. For *circHomer1* after Bicuculline and 4-Aminopyridine treatment: t = 2.767, df = 5, and p = 0.0395. In all bar graphs the individual replicates are shown within the graph.

We have previously shown that EIF4A3 can bind to *circHomer1* and positively regulate its expression, identifying EIF4A3 as an upstream regulator of *circHomer1* synthesis^35^. In addition, we recently showed that the imprinted lncRNA *H19*, which has been previously shown to bind to EIF4A3 and obstruct its recruitment to downstream RNA targets, is a negative regulator of neuronal *circHomer1* biogenesis and displays opposing developmental expression within the brain^40^. In order to identify additional factors that could modulate *circHomer1* biogenesis, we searched the literature for RBPs enriched in the brain that could bind to EIF4A3 and have either been shown to or been predicted to bind to *circHomer1*. We found that the activity-dependent RBP FUS, which has been previously linked to synaptic function, can directly bind to EIF4A3 protein^41,42^ and also has predicted binding sites for *circHomer1*. To manipulate *FUS* expression, we used two different shRNAs against human *FUS* in differentiated human SHSY-5y cells and extracted RNA for *circHomer1* and *FUS* mRNA measurements via qRT-PCR after 2days of shRNA treatment (Fig. 1A). Our results suggest that both shRNAs were able to significantly knock down *FUS* mRNA expression, which resulted in a significant downregulation of *circHomer1* levels (Fig. 1A). This effect seemed to be specific since no changes were observed in *circCDR1as* expression following *FUS* knockdown (Fig. 1A). Also, there were no changes in *EIF4A3* mRNA expression, while a modest increase in *HOMER1B* mRNA levels was observed following *FUS* knockdown (Fig. 1A). In order to determine whether FUS can directly bind to *circHomer1* we performed RIP with an anti-FUS antibody and measured *circHomer1* expression (Fig. 1B). Our data suggested that FUS does not bind directly to mature *circHomer1* (Fig. 1B). However, previous work has suggested that FUS can bind into the intronic complementary sequences necessary for circRNA backsplicing, thus promoting circRNA biogenesis^89–90^. To determine if FUS could be bound to such sequences within *pre-Homer1b*, we designed exon/intron primers that can amplify *pre-Homer1b*. Our results suggest a trend for significant enrichment for *pre-Homer1b*, as well as for *HuD* mRNA, an RBP previously known to bind directly to both FUS and *circHomer1* (Fig. 1B). This suggests the possibility that FUS not only binds to the intronic regions within *pre-Homer1b* mRNA necessary for *circHomer1* backsplicing but also binds to EIF4A3, which is close to the backspliced junction of *circHomer1*. The binding site for FUS is located on intron 5 that is necessary for the back-splicing of *circHomer1* (Fig. 1C). In addition, EIF4A3 is bound 20–24 nucleotides upstream of exon 5 (Fig. 1C), suggesting the possible binding sites to be in close proximity to synergistically facilitate *circHomer1* biogenesis.

### Neuronal activity and CREB/CBP promote circHomer1, Eif4a3, and Fus mRNA expression in mouse cortical neurons.

A previous study in mouse hippocampal neurons that were treated with bicuculline for 12 h, suggested that *circHomer1* could be activity-dependent^11^. To further test the activity-dependent nature of *circHomer1*, we decided to treat mouse primary cortical neurons with bicuculline (Bic) and 4-Amynopyridine (4-AP) for either 4, 12, or 24 hours. RNA was then extracted to measure *circHomer1, Fus*, and *Eif4a3* mRNA levels (Fig. 1D). Our results show that *circHomer1* levels were significantly upregulated 12 hours following Bic/4-AP (Fig. 1E), consistent with previous findings with in mouse hippocampal neurons with Bic only^11^. However, no changes in *circHomer1* expression were observed after 4 hours of Bic/4-AP, whereas a non-significant modest increase was seen 24 hours after treatment (Fig. 1E). Interestingly, *Fus* and *Eif4a3* mRNA levels increased significantly at just 4 hours after Bic/4-AP but were down to normal levels after 12 hours (Fig. 1E). This suggests that the activity-dependent transcription of *Fus* and *Eif4a3* mRNAs happens before the actual changes in *circHomer1* biogenesis (Fig. 1E). In a previous publication, we have shown that EIF4A3 inhibition decreases *circHomer1* levels in neurons at basal conditions^35^. To gain more insight into the mechanistic pathways of *circHomer1* biogenesis, we sought to investigate whether EIF4A3 inhibition abrogates activity dependent *circHomer1* regulation. Thus, we treated neurons with Bic + 4AP to induce neuronal activity, in the presence (or absence) of an EIF4A3 inhibitor (Fig. 1F). We replicated the findings of *circHomer1* upregulation upon synaptic activation in the absence of the inhibitor, but most importantly, we found that co-treatment with EIF4A3 inhibitor prevented the change of *circHomer1* levels in response to neuronal activity (Fig. 1F). These results were specific for *circHomer1* since no such pattern was seen for *circTulp4* (Fig. 1F), another brain enriched circRNA, allowing us to conclude that EIF4A3 inhibition abrogates *circHomer1* biogenesis both in basal and neuronal activity-dependent conditions.

**Figure 2:**
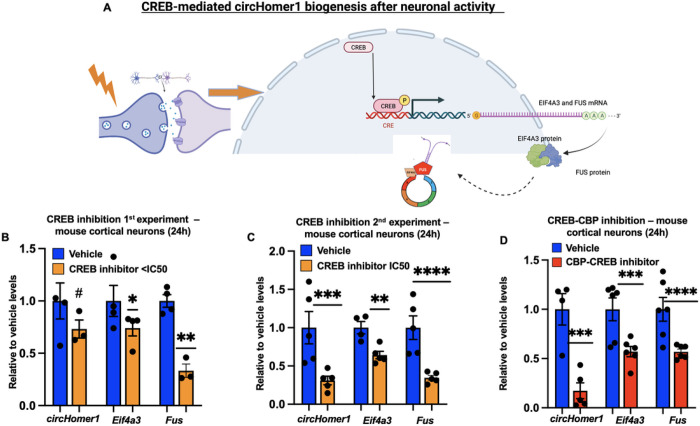
*circHomer1*is indirectly regulated by CREB stimulation. (A) Schematic connecting neuronal activity and CREB-mediated *Eif4a3/Fus* mRNA transcription for the subsequent *circHomer1* biogenesis. (B-D) Relative to vehicle, *circHomer1, Eif4a3* and *Fus* mRNA levels (quantifications were based on qRT-PCR and normalized to 18S rRNA) after treatment with CREB inhibitor (B-C) in two rounds of experiment at (B) dose < IC50 and (C) dose equal to IC50 in mouse primary cortical neurons (treatment was done for 24hours) and (D) after treatment with a CREB-CBP inhibitor at a dose equal to IC50. Mean ± SEM. ****p < 0.0001, ***p < 0.001, **p < 0.01, *p < 0.05, based on two tailed one sample t-test relative to vehicle. For *circHomer1*after treatment with < IC50 dose (B): t = 3.106, df = 2, and p = 0.0899. For *Eif4a3* mRNA after treatment with < IC50 dose (B): t = 3.441, df = 3, and p = 0.0412. For *Fus* mRNA after treatment with < IC50 dose (B): t = 10.53, df = 2, and p = 0.0089. For *circHomer1* after treatment with = IC50 dose (C): t = 12.53, df = 4, and p = 0.0002. For *Eif4a3* mRNA after treatment with = IC50 dose (C): t = 7.358, df = 4, and p = 0.0018. For *Fus* mRNA after treatment with = IC50 dose (C): t = 20.86, df = 4, and p < 0.0001. For *circHomer1* after treatment with CREB-CBP inhibitor (D): t = 10.29, df = 4, and p = 0.0005. For *Eif4a3* mRNA after treatment with CREB-CBP inhibitor (D): t = 8.238, df = 5, and p = 0.0004. For *Fus* mRNA after treatment with CREB-CBP inhibitor (D): t = 15.60, df = 5, and p < 0.0001. In all bar graphs the individual replicates are shown within the graph.

Given the fact that *circHomer1* is induced upon synaptic activity we wanted to examine which transcription regulators might be responsible for these changes. We primarily focused on cAMP-response element binding protein (CREB) element, since it has been reported to control neuronal gene expression and implicated in the pathophysiology of SCZ and BD. However, with the exception of alternative promoter CREB elements necessary for *Homer1a* transcription, no CREB-regulatory elements are present in the *Homer1* promoter responsible for the transcription of the long *Homer1* transcripts *Homer1b* and *Homer1c*^43^. Thus, the activity-dependent nature of *circHomer1* expression is unlikely to be a result of a direct transcriptional effect on *pre-Homer1b* synthesis. On the other hand, *in silico* prediction of CREB-binding sites suggests the presence of CREB-binding elements within the promoter of both *FUS* and *EIF4A3*^43^. We hypothesized that neuronal activity could induce a CREB-dependent transcriptional activation of both *Fus* and *Eif4a3* mRNAs. Upon translation of these mRNAs into proteins, we then hypothesized that Fun and Eif4a3 can synergistically promote *circHomer1* biogenesis within the nucleus (Fig. 2A). We used a CREB inhibitor to determine the effects of CREB in *circHomer1*, *Fus*, and *Eif4a3* mRNA expression (Fig. 2B–C). A low dose (< IC50) and a dose equal to the IC50 were used to determine if there is a dose-dependent effect (Fig. 2B–C). Our data show that both doses of CREB inhibitor resulted in significant reductions in *circHomer1, Fus*, and Eif4a3 mRNA expression; with the more robust changes being observed within the high dose (Fig. 2B–C). To further validate these findings, we used a CREB-binding protein (CBP) chemical inhibitor and found a significant downregulation in *circHomer1, Fus*, and *Eif4a3* mRNA expression (Fig. 2D). Of note, we also assessed the expression of known CREB-regulated neuronal genes and showed that both *c-Fos* and *Bdnf* mRNA levels were significantly downregulated after CREB inhibition (Supplementary Fig. 1A) as expected. Similarly, the activity-dependent short *Homer1a* isoform, which has a CREB-binding site in its unique promoter, was also significantly downregulated after CREB inhibition, with no changes being observed in *Homer1b* mRNA levels (Supplementary Fig. 1B). Moreover, both *c-fos* and *Bdnf* mRNA were also upregulated after just 4 hours of Bic/4-AP treatment, similar to what was observed for *Eif4a3* and *Fus* mRNAs (Supplementary Fig. 1C). This data suggests that *circHomer1, Fus*, and *Eif4a3* are activity-dependent genes regulated by the CREB/CBP pathway, and that the activity-dependent transcriptional control of *Fu*s and *Eif4a3* mRNA expression precedes the observed activity-dependent changes in *circHomer1* expression in cortical neurons.

### The PKA/MEK/ERK pathways promote circHomer1, Eif4a3, and Fus mRNA expression in mouse cortical neurons.

**Figure 3:**
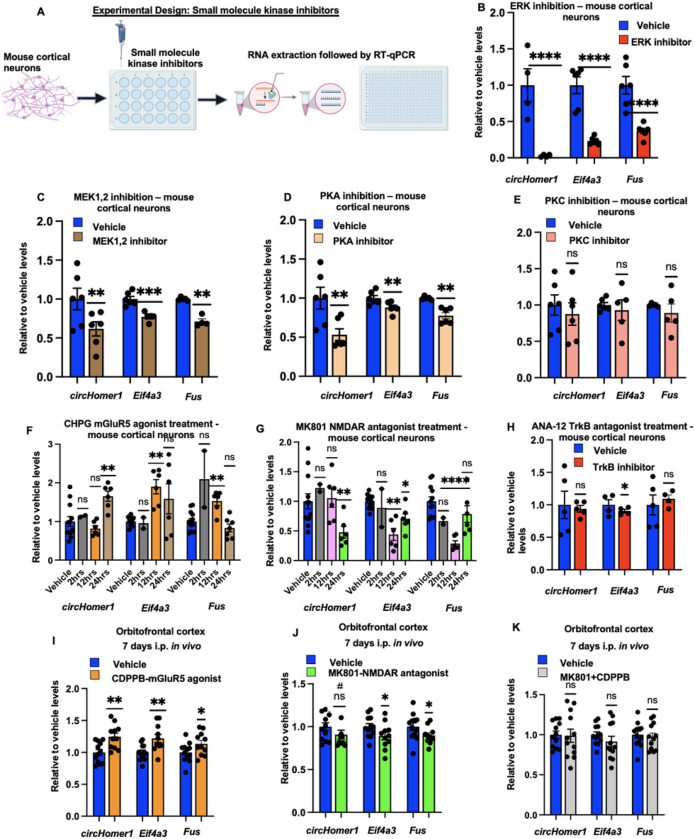
MAPK cascade, PKA and the glutamatergic system can modulate *circHomer1* and its upstream regulators (primary cortical neurons and mouse OFC) (A) Experimental Design – Primary mouse cortical neurons were plated in multiple laminin-coated 24-well plates, and on DIV13 (mature), potent and selective small molecule kinase inhibitors were added – treatments were done for 24hours. (B-E) Relative to vehicle, *circHomer1, Eif4a3* and *Fus* mRNA levels (based on qRT-PCR and normalized to 18S rRNA) after treatment with (B) ERK inhibitor (C) MEK1,2 inhibitor – kinase upstream of ERK (D) PKA inhibitor and (E) PKC inhibitor. Mean ± SEM. *p < 0.05, **p < 0.01, ****p < 0.0001, based on two tailed one sample t-test relative to vehicle. For *circHomer1* in (B): p < 0.0001, t = 107.4, df = 3. For *Eif4a3* in (B): p < 0.0001, t = 36.50, df = 5. For *Fus*in (B): p < 0.0001, t = 16.23, df = 5. For *circHomer1* in (C): p = 0.0083, t = 4.226, df = 5. For *Eif4a3* in (C): p = 0.0001, t = 8.624, df = 4 For *Fus* in (C): p = 0.0034, t = 8.539, df = 3. For *circHomer1*in (D): p = 0.0016, t = 6.220, df = 5. For *Eif4a3*in (D): p = 0.0091, t = 4.124, df = 5. For *Fus*in (D): p = 0.0027, t = 5.521, df = 5. In all bar graphs the individual replicates are shown within the graph. (F-H) Relative to vehicle *circHomer1, Eif4a3* and *Fus* mRNA levels (based on qRT-PCR and normalized to 18S rRNA) after treatment with (F) CHPG – mGluR5 agonist (treatment for 2–12-24hours), (G) MK801 – NMDAR antagonist (treatment for 2–12-24hours) and (H) ANA12 – TrkB antagonist (treatment for 24hours). Mean ± SEM. *p < 0.05, **p < 0.01, ****p < 0.0001, based on two tailed one sample t-test relative to vehicle. For *circHomer1*in (F) after 24 hours of treatment: p = 0.0040, t = 5.038, df = 5. For *Eif4a3* in (F) after 12 hours of treatment: p = 0.0047, t = 4.851, df = 5. For *Fus* in (F) after 12 hours of treatment: p = 0.0055, t = 4.666, df = 5. For *circHomer1* in (G) after 24 hours of treatment: p = 0.0032, t = 5.303, df = 5. For *Eif4a3* in (G) after 12 hours of treatment: p = 0.0025, t = 5.591, df = 5. For *Fus* in (G) after 12 hours of treatment: p < 0.0001, t = 15.38, df = 5. For *Eif4a3*in (H): p = 0.0275, t = 4.029, df = 3. (I-K) Relative to vehicle *circHomer1, Eif4a3* and *Fus* mRNA levels (based on qRT-PCR and normalized to 18S rRNA) after treatment with (I) CDPPB – mGluR5 positive allosteric modulator, (J) MK801 – NMDAR antagonist and (J) a combination of both MK801 and CDPPB as a rescue experiment – all treatments were done for 7 days. Mean ± SEM. *p < 0.05, **p < 0.01, ****p < 0.0001, based on two tailed one sample t-test relative to vehicle. For *Eif4a3* in (I): p = 0.0378, t = 2.392, df = 10. For *Fus* in (I): p = 0.0110, t = 3.190, df = 9. For *circHomer1* in (J): p = 0.0006, t = 4.708, df = 11. For *Eif4a3* in (J): p = 0.0023, t = 3.928, df = 11. For *Fus* in (J): p = 0.0178, t = 2.784, df = 11. In all bar graphs the individual replicates are shown within the graph.

Based on these findings we wanted to further dissect the intracellular molecular pathways that could regulate *circHomer1* expression in cortical neurons. We focused on the intracellular kinases that have been proposed to act upstream of CREB^44^, and we treated mouse primary cortical neurons with either ERK, MEK1/2, PKA, or PKC inhibitors for 24 hours (Fig. 3A); *circHomer1, Fus*, and *Eif4a3* mRNA expression was subsequently measured with qRT-PCR (Fig. 3A). We found that ERK inhibition resulted in a robust downregulation of *circHomer1, Fus*, and *Eif4a3* mRNA levels (Fig. 3B). Moreover, both MEK1/2 and PKA inhibition led to significant downregulation of *circHomer1, Fus*, and *Eif4a3* mRNA expression, but treatment with a PKC-specific inhibitor had no effect on *circHomer1* levels and its upstream regulators (Fig. 3C–E). This data allows us to suggest that the PKA and MEK/ERK pathways, both of which are capable of activating CREB-mediated transcription^44,45^, are positive regulators of *circHomer1* expression within cortical neurons and that *Fus* and *Eif4a3* transcriptional activation always displays similar patterns to those *circHomer1* levels.

### NMDA and mGluR5 receptors are upstream regulators of circHomer1 expression in vitro and in vivo.

Given the robust effects of ERK/CREB on *circHomer1* expression in cortical neurons, we decided to investigate the upstream neuronal receptors that activate ERK and CREB and could potentially result in an activation of *circHomer1* biogenesis. In doing so, we treated mouse cortical neurons with the mGluR5 agonist CHPG for 2, 12, and 24 hours (Fig. 3F). Our results show that *circHomer1* was significantly upregulated after 24 hours of mGluR5 activation, with no changes observed following 2 or 12 hours of treatment (Fig. 3F). Interestingly, changes in *Fus* and *Eif4a3* mRNA expression were observed at 12 hours after CHPG treatment (Fig. 3F), suggesting that transcriptional activation of these two positive regulators of *circHomer1* biogenesis precedes the increase of *circHomer1* levels. As a positive control, we also measured the expression of the activity-dependent genes *c-Fos* and *Homer1a*, both of which were found to be significantly upregulated as early as 2 hours after CHPG treatment (Supplementary Fig. 1D). We then treated mouse cortical neurons with MK801, a non-competitive NMDA antagonist, and measured *circHomer1, Fus*, and *Eif4a3* mRNA expression via qRT-PCR after 2, 12 and 24 hours (Fig. 3G). A significant downregulation in *circHomer1* expression was found after 24 hours (Fig. 3G) suggesting that NMDA receptor activation could promote *circHomer1* production within neurons. Interestingly, after 12 hours of treatment, we observed no changes of *circHomer1* levels, but a robust decrease in the mRNA levels of *Eif4a3* and *Fus* (Fig. 3G). A modest reduction in *Eif4a3* mRNA was still observed after 24 hours (Fig. 3G). This supports our proposed biogenesis model that requires the transcriptional activation of these two positive regulators for subsequent *circHomer1* induction. BDNF/TrkB signaling can also modulate ERK/CREB activation and is a potential upstream factor of circHomer1 biogenesis. Thus, we treated cortical neurons with ANA-12, a TrkB-specific antagonist (Fig. 3H). Our results suggest that TrkB receptor activation is not an upstream regulator of *circHomer1* expression in cortical neurons.

Given our findings in mouse cortical neuronal cultures, we decided to determine whether *in vivo* activation mGluR5 activation and NMDA blockade can also affect *circHomer1* expression in the mouse brain. To that end, we treated adult male WT mice with the NMDAR antagonist MK801, the mGluR5 positive allosteric modulator CDPPB, or their combination, i.p. for 7 days, after which *circHomer1, Fus*, and *Eif4a3* mRNA expression was measured in relevant brain regions using RT-qPCR.

Our results suggest that NMDA antagonism and mGluR5 activation can differentially affect *circHomer1* expression *in vivo* (Fig. 3I–K and Supplementary Fig. 2A); with MK801 treatment showing the strongest effects in the NAc and CDPPB treatment affecting *circHomer1* levels only in the OFC (Fig. 3I–J and Supplementary Fig. 2A-B). Interestingly, co-treatment with CDPPB (which normalizes increases in locomotor behavior induced by MK-801; Supplementary Fig. 2F) also normalized *circHomer1* levels in both brain regions (Fig. 3K and Supplementary Fig. 2C). As seen *in vitro*, changes in *Fus* and *Eif4a3* mRNA expression mirrored those of *circHomer1* for all treatments and in both brain regions. This further supports our hypothesis that transcriptional changes in these two RBPs are capable of promoting *circHomer1* biogenesis and are important for modulating *circHomer1* expression within the brain. Furthermore, measurements of the activity-dependent short *Homer1a* mRNA isoform in the same mice suggested that *Homer1a* mRNA was also significantly upregulated by CDBBP treatment in both the OFC and the NAc (Supplementary Fig. 2D-E). Taken together, these data suggest that NMDA blockade can reduce *circHomer1* levels within the mouse brain, while concomitant mGluR5 activation can rescue *circHomer1* expression.

### In vivo antagonism of D2 and 5HT2A receptors differentially affects circHomer1 expression in multiple brain regions.

**Figure 4:**
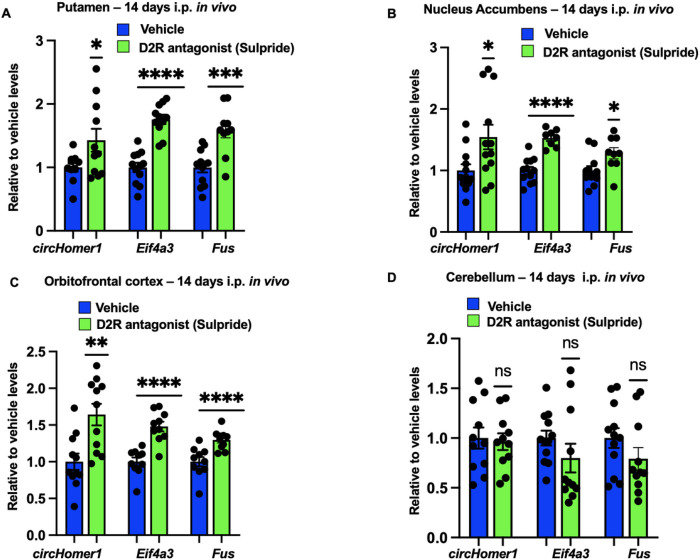
Sulpiride, pure D2R antagonist, robustly upregulates *circHomer1, Eif4a3* and *Fus* in brain regions that dopaminergic receptors are abundant. (A-D) Relative to vehicle *circHomer1, Eif4a3* and *Fus* mRNA levels (based on qRT-PCR and normalized to 18S rRNA) after treatment with Sulpiride in (A) Putamen - dorsal striatum, (B) Nucleus Accumbens – ventral striatum, (C) Orbitofrontal cortex and (D) Cerebellum. Mean ± SEM. Mice were sacrificed 24hrs after the last injection – delayed time point to clarify if the possible change of *circHomer1* levels after pharmacological intervention is persistent over time. *p < 0.05, **p < 0.01, ***p < 0.001, ****p < 0.0001, two tailed one sample t-test relative to vehicle. For *circHomer1* in (A): p = 0.0390, t = 2.375, df = 10. For *Eif4a3* in (A): p < 0.0001, t = 10.34, df = 10. For *Fus* in (A): p = 0.0008, t = 4.914, df = 9. For *circHomer1* in (B): p = 0.0199, t = 2.720, df = 11. For *Eif4a3* in (B): p < 0.0001, t = 10.63, df = 7. For *Fus* in (B): p = 0.0158, t = 3.051, df = 8. For *circHomer1* in (C): p = 0.0014, t = 4.358, df = 9. For *Eif4a3* in (C): p < 0.0001, t = 7.270, df = 9. For *Fus* in (C): p < 0.0001, t = 7.151, df = 9. In all bar graphs the individual replicates are shown within the graph.

**Figure 5:**
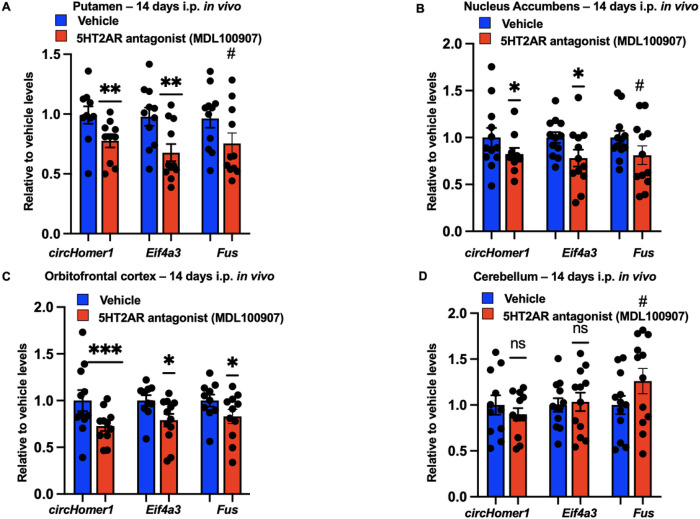
MDL100907, pure 5T2AR antagonist, robustly downregulates *circHomer1, Eif4a3*and *Fus* in brain regions that serotonergic receptors are highly present. (A-D) Relative to vehicle *circHomer1, Eif4a3* and *Fus* mRNA levels (based on qRT-PCR and normalized to 18S rRNA) after treatment with MDL100907 in (A) Putamen - dorsal striatum, (B) Nucleus Accumbens – ventral striatum, (C) Orbitofrontal cortex and (D) Cerebellum. Mean ± SEM. Mice were sacrificed 24hrs after the last injection – delayed time point to clarify if the possible change of *circHomer1* levels after pharmacological intervention is persistent over time. #0.10 < p < 0.05, *p < 0.05, **p < 0.01, ****p < 0.0001, based on two tailed one sample t-test relative to vehicle. For *circHomer1* in (A): p = 0.0030, t = 4.034, df = 9. For *Eif4a3* in (A): p = 0.0041, t = 3.606, df = 11. For *Fus* in (A): p = 0.0614, t = 2.082, df = 11. For *circHomer1* in (B): p = 0.0238, t = 2.714, df = 9. For *Eif4a3* in (B): p = 0.0366, t = 2.427, df = 11. For *Fus* in (B): p = 0.0848, t = 1.894, df = 11. For *circHomer1* in (C): p = 0.0004, t = 5.134, df = 10. For *Eif4a3*in (C): p = 0.0102, t = 3.096, df = 11. For *Fus*in (C): p = 0.0499, t = 2.229, df = 10. For *Fus*in (D): p = 0.0848, t = 1.894, df = 11. In all bar graphs the individual replicates are shown within the graph.

In addition to NMDA and mGluR5 receptors, 5HT2A and D2 neuronal receptors are of relevance to psychiatric disease due to the fact they are targets of antipsychotics^46^. Mechanistically, D2 receptors can block adenyl cyclase and inactivate the PKA/CREB molecular pathway, which is expected to result in reduced *circHomer1* expression. On the other hand, activation of 5HT2A receptors leads to phospholipase C-mediated calcium release and MEK/ERK activation^47^, which is expected to increase *circHomer1* biogenesis. Therefore, D2R antagonism is expected to disinhibit *circHomer1* expression, whereas 5HT2AR antagonism is expected to result in the downregulation in *circHomer1* expression. To test this hypothesis, we treated adult male mice with either the D2R-antagonist, Sulpiride, or the 5HT2AR antagonist, MDL100907, for 14 days. and *circHomer1, Fus*, and *Eif4a3* mRNA were measured in the putamen, NAc, OFC, ventral hippocampus, and cerebellum with qRT-PCR. Sulpiride resulted in a significant upregulation of *circHomer1, Fus*, and *Eif4a3* mRNA levels in the OFC, NAc and Putamen, all of which are brain regions that express D2R^48,49^ (Fig. 4A–C). However, no changes were observed within the cerebellum (Fig. 4D), a brain region with very little D2R expression. On the other hand, treatment with 5HT2AR antagonist MDL100907 resulted in a significant downregulation of *circHomer1, Fus*, and *Eif4a3* mRNA levels in the OFC, NAc and putamen (Fig. 5A–C), all of which are brain regions that also express 5-HT2AR^50,51^. Similarly, to what was observed with Sulpiride, 5-HT2AR antagonism had no effect on *circHomer1, Fus*, and *Eif4a3* mRNA levels within the cerebellum, a brain region that is also devoid of 5HT2AR (Fig. 5D). Moreover, a reduction in *circHomer1, Fus*, and *Eif4a3* mRNA levels was also found in the ventral hippocampus after 5HT2AR but not D2R antagonism, which is expected given the high expression of 5HT2AR and low expression of D2R in that brain region^52–55^ (Supplementary Fig. 3A-B). This further supports our hypothesis that changes in *Fus* and *Eif4a3* transcription are necessary for the modulation of *circHomer1* biosynthesis. We conclude that D2 and 5HT2A receptors differentially modulate *circHomer1, Fus*, and *Eif4a3* mRNA expression within multiple brain regions that are linked to SCZ and BD pathogenesis. Dopamine D2AR blockade increases *circHomer1* and its upstream regulations, whereas 5HT2AR blockade reduces *circHomer1, Eif4a3* and *Fus* in multiple mouse brain regions.

### Treatment with Olanzapine differentially affects circHomer1 expression in cortical and subcortical regions in vivo.

Based on the literature, all antipsychotics block D2 receptors and several, among which the widely used second generation “atypical” antipsychotic^56^ olanzapine, also block 5HT2R receptors^57,58^. Given our above findings showing a bidirectional effect of D2 and 5HT2A receptor blockade on *circHomer1* expression, we thought to evaluate the effects of olanzapine on the biosynthesis of circHomer1 in cortical and subcortical brain regions. Mice were treated with olanzapine, (at an effective dose that reduced locomotor activity; Supplementary Fig. 3C) for 14 days. Subsequently, the putamen, NAc, OFC, and cerebellum were extracted for qRT-PCR quantification of *circHomer1, Fus,* and *Eif4a3* mRNA. We show that that *circHomer1* was not altered following olanzapine treatment in the putamen and NAc (Supplementary Fig. 4A-B), whereas a 23% reduction was observed within the OFC (Supplementary Fig. 4C), possibly due to the higher presence of 5HT2A than D2 receptors in this specific brain region. A significant downregulation was observed in the cerebellum, a brain region that does not have considerable expression of D2 and 5HT2A expression, but is known to be enriched in other receptors, such as the muscarinic and cholinergic, that can be targeted by olanzapine^56,59^ (Supplementary Fig. 4D). This also could potentially feed mechanistically into the same regulatory pathways responsible for *circHomer1* biogenesis.

## DISCUSSION

Recent studies have shown that circRNAs are enriched in the brain, expressed in the synapses, and preferentially generated from synaptic-related genes^8,9,11^, as is the case for *circHomer1*, discussed in detail above. Despite recent advances regarding the role of circRNAs in regulating brain function and their implications for the pathophysiology of brain disorders^12,13,60,61^, the molecular mechanisms that underlie the biogenesis of these novel circular noncoding regulators in general and *circHomer1* in particular, remain underexplored.

**Figure 6:**
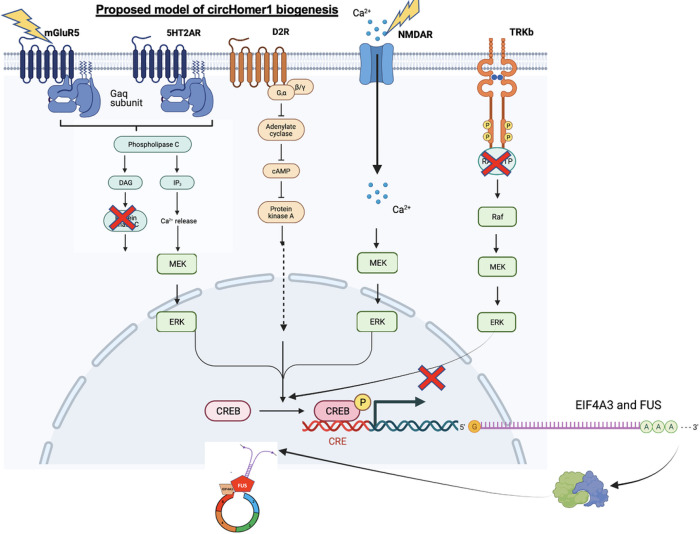
Proposed schematic of *circHomer1* regulation, summarizing the findings of this study. Following synaptic activity and postsynaptic neuronal excitation, the metabotropic glutamate receptors mGluR5 and the ionotropic glutamate receptors NMDAR will allow intracellular Ca+2 levels to be elevated either from the endoplasmic reticulum (ER) or by directly entering from the plasma membrane. This Ca+2 elevation will activate the downstream protein kinase ERK1/2, which in turn will phosphorylate and activate CREB. 5HT2AR that share the same signaling pathway with mGluR5 (G_aq_ subunit) will also stimulate ERK1,2 and lead to subsequent CREB activation. In addition, the dopaminergic system via the D2R signaling pathway and regulation of PKA, can modulate the CREB-mediated transcription of activity dependent genes. Notably, the glutamatergic and the dopaminergic systems are well-known to be dysregulated in SCZ and BD subjects. Following the CREB-mediated neuronal activity dependent mRNA transcription of *EIF4A3* and *FUS*, these mRNAs will be translocated to the cytosol for protein translation, will then enter to the nucleus, and act as RBPs promoting *circHomer1* biogenesis. Conversely, the BDNF-TrkB pathway, that acts as a negative feedback loop for CREB modulation, is not shown to be implicated in *circHomer1* biogenesis.

This work provides insights into the molecular circuits that underlie *circHomer1* biogenesis in the brain and its regulation by neurotransmitter systems implicated in psychiatric diseases namely SZC (Fig. 6). We demonstrate that EIF4A3 (one of the RBPs that acts upstream of *circHomer1*^35^) is necessary for the neuronal- activity-dependent regulation of *circHomer1* biogenesis. We have previously shown that *EIF4A3* mRNA expression in human postmortem brains is significantly downregulated in the OFC of BD patients and is positively correlated with the relative changes in OFC *circHomer1* expression. Here we provide evidence of a causal effect. We show that *circHomer1* can be induced upon neuronal activity in an EIF4A3 manner since treatment of neuronal cultures with Bic + 4AP upregulated circHomer1 levels, an effect that was abolished by pretreatment with EIF4A3 inhibitor. Other than EIF4A3, FUS is a nuclear ribonucleoprotein that has been implicated in pre-mRNA splicing, by virtue of binding to nascent pre-mRNA, and is shown to play crucial roles in dendritic spine formation, mRNA stability, and synaptic homeostasis^62,63^. Since FUS can directly bind to EIF4A3, we hypothesized FUS to also facilitate the back-splicing of *circHomer1*. FUS binds to intronic regions without a sequence specificity, although showing a preference for GU-rich regions^64,65^. Despite this, the data obtained from the RIP experiment agrees with the current literature, as they point towards a mechanism where *Homer1b pre-mRNA*, but not mature *circHomer1* can directly bind to FUS. Notably, by using multiple shRNAs capable of targeting *FUS* mRNA, we have also shown that knockdown of *FUS* can result in a reduction of *circHomer1* and that *FUS* mRNA changes are positively correlated with the relative changes in *circHomer1* expression.

CREB is a transcriptional activator of activity-dependent gene expression implicated in long-term synaptic potentiation and neuronal plasticity^66,67^ and linked to the pathophysiology of psychiatric disorders including SCZ^68^. Here we demonstrate that CREB is a positive regulator of *circHomer1* biogenesis that could act by affecting the transcription levels of *circHomer1* upstream regulators *EIF4A3* and *FUS*, both of which have CREB elements on their promoters. Notably, CBP, which has been characterized as a transcriptional coactivator^69^, was also found to modulate *circHomer1* levels since. Indeed, we showed that treatment with a CREB inhibitor and CREB-CBP inhibitor in mouse cortical neurons resulted in a significant downregulation in *circHomer1, Fus*, and *Eif4a3* mRNA expression. Since CREB is activated upon neuronal excitation, we treated primary neuronal cultures with Bicuculline, (a GABAa antagonist), and 4-Aminopyridine, (a voltage K channel blocker), which combined can lead to substantial neuronal activation; we showed that all *Eif4a3, Fus*, and *circHomer1* are induced in an activity-dependent manner.

According to our hypothesis, after synaptic activity and stimulation, CREB would be activated and translocated to the nucleus, thus promoting the transcription of *Eif4a3* and *Fus* mRNA, which in turn would facilitate *circHomer1* biogenesis. From the literature, it is well established that transcription is performed in the nucleus of the cell, and the mRNA must then be trafficked to the cytoplasm for subsequent protein translation^70–72^. A protein that possesses a nuclear localization signal will then enter the nucleus. This cellular trafficking may provide some explanation for the differences in temporal regulation as described earlier, mainly the increase in *Eif4a3* and *Fus* mRNA levels 4 hours after neuronal activation and the subsequent increase of *circHomer1* 8 hours later (12hrs after Bicuculline and 4-Aminopyridine treatment). Interestingly, a similar difference in the timing of *Eif4a3* and *Fus* mRNA and *circHomer1* induction was observed following treatment of cortical neurons with an mGluR5 agonist and NMDAR antagonist. Of note, to further strengthen this proposal, further experimentation such as ChIP should be done to confirm that CREB/CBP directly binds to the *Eif4a3* and *Fus* promoters to induce their expression in primary cortical neurons.

It has been proposed in the literature that different protein kinases can phosphorylate and activate CREB, making it a common target of multiple intracellular signaling cascades^73,74^. Namely, PKA activates CREB by phosphorylating the amino acid residue Ser133 in the KID domain via the canonical signaling pathway. Of note, there is evidence that PKA not only can activate CREB directly, but also indirectly via the non-canonical pathway and phosphorylation of MEK^44^. MEK, also known as MAPKK, is part of the MAPK/ERK signaling cascade, which is reported to be crucial for synaptic plasticity and long-term potentiation. Here, we wanted to identify the kinases implicated in *circHomer1* biogenesis. We provide evidence that the MAPK signaling cascade, along with PKA can elevate the transcription of *Eif4a3* and *Fus* mRNA and thus, lead to the downstream EIF4A3/FUS-mediated increase in *circHomer1* biogenesis. From our data, after inhibiting MEK, a reduction of *circHomer1* and its upstream regulators was observed in primary neuronal cultures. In parallel, inhibition of the major executive kinase of the MAPK pathway in neurons, the extracellular signal-regulated kinase ERK, diminished *circHomer1* levels in the cortical neurons.

We next sought to investigate neurotransmitter systems that could regulate the *circHomer1* biogenesis upstream of PKA and ERK. We specifically chose to target 5HT2AR and D2R.receptors, since *circHomer1* is a SCZ-related circRNA and since second-generation antipsychotics -the current treatment for SCZ- have a multi-receptor targeting profile, yet most of them act on the 5HT2AR and D2R. After subchronic (14 days) administration in mice, MDL100907- selective 5HT2AR antagonist robustly decreased *circHomer1* levels and the mRNA levels of *Eif4a3* and *Fus* in brain regions that are highly associated with SCZ pathogenesis (OFC, Nucleus Accumbens and Putamen). On the contrary, sulipiride - a D2R antagonist elevated *circHomer1* levels and the mRNA of *circHomer1* upstream regulators in all three brain SCZ-related regions mentioned above. Sulpiride, a selective D2R antagonist, is already approved and on the market in the European Union for treatment of SCZ^75^. Interestingly, sulpiride has been shown to improve some of the negative symptoms such as alogia, anhedonia, apathy and eventually some cognitive symptoms^76^. Based on the increase of *circHomer1* in the OFC and the striatum after sulpiride treatment, it would be tempting to hypothesize a link between the therapeutic effects of sulpiride and the increase of *circHomer1*.

Intriguingly, olanzapine, one the most widely used second generation antipsychotics with a preferential affinity for D2 and 5HT2A receptors, did not lead to robust changes of *circHomer1* levels in the mouse striatum, with a 23% reduction in the mouse OFC, a brain region that 5HT2A receptors are more abundant than D2. However, from this experiment, another interesting insight was gained: olanzapine robustly decreased *circHomer1* levels in the mouse cerebellum. Olanzapine is a drug that possesses multiple receptor targeting profile having an affinity for serotonergic 5HT2, dopaminergic D1-D2, muscarinic M1–5, and histaminic H1-H3^56,59,77^. The cerebellum is a brain region where the acetylcholinergic system is predominant, and all five isoforms of the muscarinic receptors M1–5 are highly expressed in comparison with other brain regions. Since 5HT2AR and D2R appear to be absent or scarce in the cerebellum^49,50^, we speculate a possible role for this atypical agent in *circHomer1* modulation via the cholinergic system. Further studies are needed to examine this and to understand its functional significance regarding therapeutic or side-effects of olanzapine.

On the contrary, we show that olanzapine has little, if any, effects on *circHomer1* levels in the striatum, nucleus accumbens and cortex. To explain this finding and get insights by looking at the mechanistic interpretation of the data, 5HT2AR activation affects the release of intracellular Ca + 2, which activates the MAPK cascade and the CREB phosphorylation^78^, finally promoting *circHomer1* biogenesis. Activation of the D2R signaling pathway, may inhibit adenylate cyclase and PKA, resulting in the suppression of CREB^79^ and blockade of *circHomer1* biogenesis. Therefore, olanzapine effects on *circHomer1* are blunted due to the opposing mechanistic downstream actions of D2 and 5HT2A. It is tempting to suggest that the little or zero net-effect of olanzapine on circHomer1 levels might serve as an explanation for the lack-of or little cognitive benefit with olanzapine and second-generation antipsychotics in general^80^. It is also tempting to suggest a key-role of *circHomer1* in SCZ-related signaling, as it is able to integrate information from both DA- and HT- neurotransmitter systems implicated in psychosis.

Beyond monoamines, ERK and PKA are downstream target of the glutamatergic system; it can be stimulated by excitatory signaling in particular after the activation of NMDA and mGluR5 receptors, which interact among them and with proteins of the Homer family to regulate cognitive function^81–83^. Both receptors have been implicated in the glutamatergic hypothesis of SCZ^84,85^. NMDAR hypofunction has been linked with SCZ by multiple postmortem, genetic, and pharmacological studies, and has been shown to lead to impairments in cognitive processes^86,87^. On the other hand, mGluR5, was shown to be relevant to the SCZ pathophysiology, with mGluR5 Positive Allosteric Modulator PAM having a therapeutic potential in SCZ preclinical models, particularly improving the cognitive dysfunction^88^. Given the fact that mGluR5 and NMDA receptors are highly expressed in the frontal cortex, a region that *circHomer1* has been reported to be particularly abundant^89^, one could hypothesize that *circHomer1* might be another downstream target of the neuronal transcriptional events that are initiated following activation of these receptors. Indeed, here we are providing the first ever evidence in literature that *circHomer1* biogenesis is linked to the activation of the glutamatergic system. In neuronal cell culture experiments, application of mGluR5 agonist led to an increase of *Eif4a3* and *Fus* mRNA levels and a subsequent upregulation of *circHomer1* levels. Notably, *in vivo* studies were able to replicate the *in vitro* mGluR5 data. CDPPB, an mGluR5. PAM that is brain penetrant and has been proposed to have antipsychotic action in animal models^82,90,91^ was administered to WT mice, resulting in elevated *circHomer1* levels in the OFC. On the other hand a blockade of NMDAR with MK801 -a non-competitive antagonist^92,93^- decreased *circHomer1* levels *in vitro*, a result that we were able to replicate *in vivo*, albeit not all brain regions examined. Remarkably, a concurrent administration of mGluR5 PAM (CDPPB) and NMDAR antagonist (MK801) rescued the observed alterations in *circHomer1* levels and/or the expression of its upstream regulators (*Eif4a3* and *Fus*) in the OFC and the nucleus accumbens.

Given the notion that *circHomer1* is downregulated in the DLPFC and OFC of SCZ and BD subjects and *circHomer1* role in cognitive flexibility^34,35^ it is tempting to suggest that an increase of *circHomer1*, as seen with mGluR5 activation, would be a novel approach to improve cognitive symptoms. We show here that a mGluR5 PAM increased *circHomer1* levels and also rescued the reduced *circHomer1* levels after MK801 administration (a mouse model that can mimic predominantly the cognitive symptoms of SCZ). Previous literature supports the notion that in the same SCZ-like mouse model, administration of mGluR5 improved the cognitive impairments^94,95^. Multiple preclinical studies in a variety of animal models of neuropsychiatric disorders have demonstrated antipsychotic like properties of mGluR5 PAMs^96–98^. In addition, in the MK801 rodent model, behaviors associated with cognition were reversed by CDPPB^90,91^. However, these agents are still under investigation in clinical trials and there is still room for improvement^99^.

It has been implied that the primary function of the DLPFC is to regulate executive function, which is dysregulated in SCZ. OFC regulates the executive function and cognitive flexibility, which is impaired in both SCZ and BD subjects, and is hard to improve using current therapeutics. Our lab’s recent publications have shown behavioral deficits in the reversal learning paradigm after *circHomer1 in vivo* KD, suggesting that the dysregulation of *circHomer1* in SCZ and BD patients could be associated with some of the cognitive disturbances. Taken together, our findings suggest that restoring *circHomer1* levels could be used as an approach to improve the treatment-resistant cognitive symptoms of psychotic disorders.

Further research is needed to understand how our results can be translated into clinical effective strategies with an unprecedented mechanism that targets circHomer1 rather than Glu receptors. Since there are many genes known to be associated with SCZ^100,101^, the possibility of other circRNAs derived from such SCZ GWAS-linked genes should be examined. Regarding the role of *circHomer1* in the regulation of SCZ-GWAS genes, our lab has previously demonstrated that in vivo circRNA-specific knockdown of *circHomer1* in mouse PFC, modulates the expression of numerous alternative mRNA transcripts from genes involved in synaptic plasticity and psychiatric disease, attributing an upstream role for the SCZ-GWAS differential expression. Lastly, mechanistic experiments that include *in vivo* manipulations of *circHomer1* via shRNA or the use of the recently developed *circHomer1* KO mice will be required in the future to solidify the importance of this circRNA as a first-in-class novel target for cognitive function in psychiatric disease.

Cognitive impairments in psychiatric disorders, SCZ in particular are still a huge unmet medical need. We hypothesized that the newly characterized circHomer1 an activity dependent and SCZ-related regulator of synaptic plasticity could provide a novel target with an innovative potential. For this it is crucial to unravel the systems that govern circHomer1 regulation. Here, we provide first insights into the intracellular circuits that underlie *circHomer1* biogenesis in the brain and mechanistic proof that targeting receptors relevant to SCZ, and antipsychotic action modify *circHomer1* expression. Overall, our data points to a mechanism linking neuronal receptor activation to ERK-PKA/CREB-mediated transcription of *Eif4a3* and *Fus* mRNAs and subsequent EIF4A3 and FUS-mediated *circHomer1* biogenesis. We also show that circHomer1 is uniquely positioned to integrate D2, 5HT2A, and glutamatergic signals in SCZ related brain regions. This study is the first to elucidate how current and under-development therapeutic agents can modulate a psychiatric disease-associated circRNA, and to propose *circHomer1* as a novel target for cognitive impairment in SCZ.
